# Nutritional Risk in Major Abdominal Surgery: Protocol of a Prospective Observational Trial to Evaluate the Prognostic Value of Different Nutritional Scores in Pancreatic Surgery

**DOI:** 10.2196/resprot.4567

**Published:** 2015-11-16

**Authors:** Pascal Probst, Sebastian Haller, Colette Dörr-Harim, Thomas Bruckner, Alexis Ulrich, Thilo Hackert, Markus K Diener, Phillip Knebel

**Affiliations:** ^1^ University of Heidelberg Department of General, Visceral and Transplantation Surgery Heidelberg Germany; ^2^ University of Heidelberg The Study Center of the German Surgical Society (SDGC) Heidelberg Germany; ^3^ Institute of Medical Biometry and Informatics University of Heidelberg Heidelberg Germany

**Keywords:** Diagnosis Related Group system, malnutrition, nutritional assessment, nutritional score, pancreatic surgery

## Abstract

**Background:**

The influence of patients’ preoperative nutritional status on their clinical outcome has already been proven. Therefore, patients with malnutrition are in need of additional therapeutic efforts. However, for pancreatic surgery, evidence suggesting the adequacy of existing nutritional assessment scores to estimate malnutrition associated with postoperative outcome is limited.

**Objective:**

The aim of the observational trial “Nutritional Risk in Major Abdominal Surgery (NURIMAS) Pancreas” is to prospectively assess and analyze different nutritional assessment scores for their prognostic value on postoperative complications in patients undergoing pancreatic surgery.

**Methods:**

All patients scheduled to receive elective pancreatic surgery at the University Hospital of Heidelberg will be screened for eligibility. Preoperatively, 12 nutritional assessment scores will be collected and patients will be assigned either at risk or not at risk for malnutrition. The postoperative course will be followed prospectively and complications according to the Clavien-Dindo classification will be recorded. The prognostic value for complications will be evaluated for every score in a univariable and multivariable analysis corrected for known risk factors in pancreatic surgery.

**Results:**

Final data analysis is expected to be available during Spring 2016.

**Conclusions:**

The NURIMAS Pancreas trial is a monocentric, prospective, observational trial aiming to find the most predictive clinical nutritional assessment score for postoperative complications. Using the results of this protocol as a knowledge base, it is possible to conduct nutritional risk-guided intervention trials to prevent postoperative complications in the pancreatic surgical population.

**Trial Registration:**

germanctr.de: DRKS00006340; https://drks-neu.uniklinik-freiburg.de/drks_web/navigate.do?navigationId=trial.HTML&TRIAL_ID=DRKS00006340 (Archived by WebCite at http://www.webcitation.org/6bzXWSRYZ)

## Introduction

### Existing Evidence and Need for a Trial

Malnutrition is estimated as one of the leading causes for loss of health [1]. For hospitalized patients, the direct negative impact of malnutrition has broadly been examined [2-7]. Patients with tumorous diseases as well as patients being treated in intensive care units or in geriatric hospitals are mostly affected by negative impact of malnutrition [8-11].

To detect malnutrition, several scores have been developed. A recently published systematic review with meta-analysis investigated 32 scores with regard to their validity and predictive value for the population of hospitalized patients. The review indicated that only a small portion of scores had been fully validated and in particular, only limited scores are available for surgery. Development of new scores was considered redundant and they were not able to achieve higher sensitivity or specificity. Thus, trials investigating different scores in a specific patient population have been claimed necessary [12].

The population of surgical patients is specifically at high risk for being malnourished [13]. For some surgical indications, malnutrition has been proven as a risk of postoperative complications [14-16]. Regarding pancreatic surgery, limited data are available due to insufficient sample sizes or inhomogeneous populations. For example, the most recent pancreas-specific trial showed a correlation between the nutritional risk index and wound infections in patients after pancreaticoduodenectomy [17]. In addition, in this trial, the small sample size of 64 patients represents the major limitation.

### Aim of the Trial

“Nutritional Risk in Major Abdominal Surgery (NURIMAS) Pancreas” (DRKS00006340) is a monocentric, prospective, observational trial with one study arm. The aim of this trial is to find the best suitable clinical nutritional assessment score to predict postoperative complications in patients undergoing pancreatic surgery.

## Methods

### Study Population

The study population will comprise adult patients undergoing pancreatic surgery at the Department of General, Visceral and Transplantation Surgery at the University Hospital of Heidelberg. All underlying diseases leading to a primary pancreatic resection will be included. Thus, the analysis will give information on a broad and representative population as seen in high-volume surgical centers (Textbox 1).

**Eligibility criteria.**

Inclusion criteria

- Age ≥ 18 and ≤ 90 years
- Elective pancreatic surgery
- Written informed consent

Exclusion criteria

- Any former pancreatic-surgical procedures
- Language problems
- Inability to understand the trial

### Diagnostic Intervention (Nutritional Assessment Scores)

Based on the most recent systematic review about existing nutritional assessment scores by Van Bokhorst-de van der Schueren [12], 11 scores have been selected that are in use in surgical patient populations [18-27]. Recently, the European Society of Clinical Nutrition and Metabolism (ESPEN) published a new consensus definition of malnutrition, the ESPEN malnutrition criteria [28]. Table 1 presents a summary of the 12 nutritional assessment scores that will be evaluated.

**Table 1:** Nutritional assessment scores.

Name	Classification for nutritional risk^a^
Nutritional Risk Index [18]	Normal/mild/moderate/severe
Nutritional Risk Screening Score and Revised Version [19,26]	Low/ moderate/high
Subjective Global Assessment [20]	No/ moderate/severe
Malnutrition Universal Screening Tool [21]	Low/ medium/high
Mini-Nutritional Assessment and Revised Version [22,27]	Normal/ at risk/malnourished
Short Nutritional Assessment Questionnaire [23]	Low/ moderate/severe
Imperial Nutritional Screening System I [24]	Not at risk/at risk
Imperial Nutritional Screening System II [24]	Green/amber/red
Nutritional Risk Classification [25]	Low/at risk
ESPEN Malnutrition Criteria [28]	Normal/malnourished

^a^The highest class for nutritional risk determined by the scores will be used as the study end point “at risk for malnutrition” for statistical evaluation.

### Outcome Measures

The primary end point is postoperative morbidity and mortality. The most suitable score is defined as the score with the highest association of malnutrition and postoperative complication expressed as the highest lower bound of the 95% confidence interval of odds ratio.

Secondary end points are length of hospital stay, length of stay in intensive care unit, comprehensive complication index [29], place of discharge (discharge to home or discharge to rehabilitation or care facility), necessity of postoperative parenteral or enteral nutrition, and impact of malnutrition as diagnosis on hospital costs and Diagnosis Related Group (DRG) case cost.

### Trial Site and Sample Size Calculation

The trial will be conducted at the Department of General, Visceral and Transplantation Surgery at the University Hospital of Heidelberg. Prevalence of malnutrition in pancreatic cancer is known to be 88% [30]. We calculated sample size with a lower prevalence of 70% for all pancreatic diseases. With a specificity and sensitivity of 95% and a confidence interval of 0.05, a total of 260 patients will be needed [31]. Patients will be consecutively recruited until the study population will consist of 260 patients with major pancreatic resections (pancreaticoduodenectomy, distal pancreatic resection, or total pancreatectomy). Based on the department’s data (about 500 eligible pancreatic resections), recruitment will be completed within 12 months after inclusion of the first patient.

### Planned Study Conduct and Trial Visits

All patients visiting the Department of General, Visceral and Transplantation Surgery at the University Hospital of Heidelberg and scheduled to receive elective operations will be screened. Eligible patients will be consecutively informed about the study purpose and conduct. After giving a written informed consent, patients will be questioned and examined (Visit 1) according to the investigated nutritional assessment scores (Table 2). Further, other known risk factors for postoperative complications will be noted [32,33]. If the operation is delayed for any reason, patients will be re-evaluated as long as preoperative data from questionnaires are not older than 36 hours at the time of actual operation.

**Table 2:** Flowchart of the NURIMAS trial-course of examinations.

Visit	1	2	3	4
	Preoperative	POD 3-7	POD 10-14	Discharge or POD 30
Eligibility	X			
Informed consent	X			
Baseline data	X			
Nutritional scores	X			
Laboratory analyses	X	x	x	x
Assessment of surgical procedure		x		
Assessment of complications		x	x	x
Serious adverse events		x	x	x
Secondary end points		x	x	x

After the operation, the clinical course will be followed prospectively. Therefore, 3 planned visits will be performed. The first visit will be performed on postoperative days (PODs) 3-7, the second visit on PODs 10-14, and the last visit on the day of discharge or not later than POD 30. During these visits, complications according to Textbox 2 [34-41] will be assessed. Every postoperative complication will be rated according to the validated classification by Clavien-Dindo [42]. Further, on postoperative visits, the status of secondary end points will be evaluated.

**Assessed postoperative complications.**

• Postoperative pancreatic fistula [34]

• Bile leak [35]

• Postpancreatectomy hemorrhage [36]

• Delayed gastric emptying [37]

• Surgical site infection [38]

• Other infections and sepsis [39]

• Chylous ascites (triglycerides in drainage) [40]

• Serious adverse event [41]

### Data Management and Monitoring

All required information according to this protocol will be recorded on a paper-based case report form. After the last visit, data will be entered in a password-protected and validated relational database (SQL Server 2008 Express). After the last patient’s last visit, database will be soft-locked. A monitoring will be performed on 100% of data necessary to evaluate the primary end point. Of the remaining data, 20% are randomly selected. Finally, the database will be closed and made available for statistical analysis.

### Statistical Analysis

**Figure 1:**
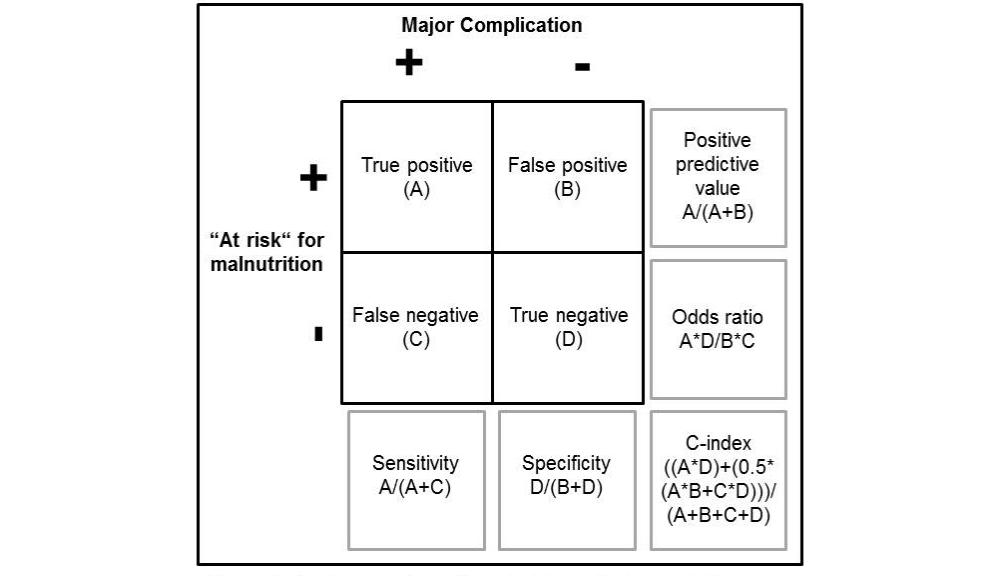
Contingency table for calculation of primary study endpoint for the prognostic value of every nutritional assessment score.

The included scores use different numbers of nutritional risk classes. To compare the scores, evaluation of the primary end point patients will be dichotomized by each nutritional assessment score as “at risk” or “not at risk” using the respective highest nutritional risk determined by each score (Table 1). Further, patients will be dichotomized whether they had at least one major complication (Clavien-Dindo III-V) or not. Hence, for every nutritional assessment score, a contingency table will be created (Figure 1). Positive predictive value, specificity, sensitivity, and c-index will be calculated. Association between every nutritional assessment score and major complication will be expressed as odds ratio with 95% confidence interval. Univariable significance of association will be tested with a chi-square test without Yate’s correction at a level of significance of 5%. A multivariable logistic regression model will be used for evaluation of primary end point at a level of significance of 5%. Covariates will be age (years) and operation time (minutes). Factors will be malignancy; gender; laparoscopy; intraoperative radiotherapy; resection of vessels (portal vein, superior mesenteric artery, or vein); inclusion in an interventional trial; American Society of Anaesthesiologists (ASA) physical status classification system; prior upper gastrointestinal surgery; pancreatic surgery associated risks (amylase in drainage >5000 IE/U on POD 1, biliary stent); and preoperative serum albumin level less than 35 g/L. Subgroup analysis will be performed separately for different types of pancreatic resections and different nutritional risk classes will be determined by each score (Table 1).

Secondary end points will be analyzed descriptively by tabulation of the measures of the empirical distributions. According to the level of the variables, means, SDs, medians, 1st and 3rd quartiles, minimum and maximum, or absolute and relative frequencies will be reported, respectively. Descriptive *P* values of the corresponding statistical tests and associated 95% confidence intervals will be given. Statistical analysis will be performed with program R [43].

### Methods for Minimizing Bias

#### Minimizing Selection Bias

All patients will be consecutively screened and if found to be eligible, informed consent will be obtained in the single-arm study. Number of screened, included, and analyzed patients will be reported and differences will be explained.

#### Minimizing Performance and Detection Bias

Preoperative data capturing and outcome assessment will be performed by 2 different investigators. Statistical analysis will be performed after closure of database.

#### Minimizing Attrition Bias

Statistical measurements such as imputation will be taken to minimize risk of bias due to incomplete outcome data [44]. The trial will be reported according to the Standards for Reporting of Diagnostic Accuracy (STARD) statement [45]. The trial is registered with Deutsches Register Klinischer Studien (DRKS00006340). To avoid the risk of selective reporting, the trial protocol with full information about end points and profound explanation of planned statistical analysis is hereby published according to the Standard Protocol Items: Recommendations for Interventional Trials (SPIRIT) statement where appropriate [46]. Report on cost issues and validation of the ESPEN criteria for malnutrition is planned separately.

#### Minimizing Other Bias

Any financial relationship or any conflict of interest that could inappropriately influence the work within this project will be stated explicitly. Confounding will be minimized by inclusion of covariates and factors in the statistical analysis of the primary end point as mentioned in the “Statistical Analysis” section.

### Ethics and Informed Consent

The NURIMAS Pancreas trial is conducted in accordance with the Declaration of Helsinki in its actual version [47]. According to the professional code for physicians in Germany (§15 BOÄ), the trial protocol was reviewed and approved by the Ethics Committee of the medical faculty of the University of Heidelberg.

Before inclusion in the NURIMAS Pancreas trial, patients will be informed both orally and in writing about all relevant aspects of the trial (eg, the aims, methods, the anticipated benefits, potential risks of the study, and the discomfort it may entail). The patients’ free decision to participate will be documented by signature on the informed consent form. All patient-related information is subject to medical confidentiality and to the Federal Data Protection Act. Pseudonymized data transfer will be performed. Third parties will not have any insight into original data.

## Results

Final data analysis is expected to be completed during Spring 2016.

## Discussion

The NURIMAS trial is a monocentric, prospective, observational trial aiming to find the most suitable clinical nutritional assessment score to predict major postoperative complications associated with malnutrition. Thus, an important lack of knowledge in preoperative risk assessment in patients undergoing pancreatic surgery will be worked-off. Upon this knowledge, further trials can rely on a validated nutritional risk and evaluate the benefit of nutritional interventions potentially preventing postoperative complications.

## Abbreviations

ASA: American Society of Anaesthesiologists
DRG: Diagnosis Related Group
ESPEN: European Society of Clinical Nutrition and Metabolism
IMBI: Institute of Medical Biometry and Informatics
POD: postoperative day
SAE: serious adverse events
SDGC: Study Center of the German Surgical Society
SPIRIT: Standard Protocol Items: Recommendations for Interventional Trials
STARD: Standards for Reporting of Diagnostic Accuracy
